# A predefined sulphur-handling transcriptional programme is associated with colorectal cancer molecular subtypes

**DOI:** 10.3389/fonc.2026.1976885

**Published:** 2026-10-16

**Authors:** Shaheryar Shafi, Arslan Hamid, Jiao Yang, Xiaoxia Cheng, Minxuan Sun

**Affiliations:** 1 School of Biomedical Engineering (Suzhou), Division of Life Sciences and Medicine, University of Science and Technology of China, Hefei, China; 2 Suzhou Institute of Biomedical Engineering and Technology, Chinese Academy of Sciences, Suzhou, China; 3 Universitätsklinikum Bonn Medizinische Klinik und Poliklinik II, Bonn, Germany; 4 Suzhou Research Center of Medical School, Suzhou Hospital, Affiliated Hospital of Medical School, Nanjing University, Suzhou, China; 5 Department of Clinical Laboratory, Suzhou Hospital, Affiliated Hospital of Medical School, Nanjing University, Suzhou, China

**Keywords:** colorectal cancer, consensus molecular subtype (CMS), SLC7A11, sulphur metabolism, transcriptional programme

## Abstract

**Background:**

Colorectal cancer (CRC) exhibits substantial metabolic and transcriptomic heterogeneity, but the relationship between sulphur-handling transcriptional programmes and consensus molecular subtypes (CMS) remains incompletely resolved.

**Methods:**

We analysed a predefined 14-gene sulphur-handling panel across The Cancer Genome Atlas colon and rectal adenocarcinoma cohorts (TCGA-COAD/READ) and six independent HG-U133 validation cohorts (*n* = 1,337 tumours). Analytical workflows integrated differential expression comparing tumours with adjacent-normal mucosa, CMS association modelling, random-effects meta-analysis, adjustment for three orthogonal tumour-purity estimators (ESTIMATE, consensus purity estimates, and ABSOLUTE), reference-based single-cell deconvolution with independent cross-atlas verification, and direction-aware therapeutic triage.

**Results:**

Colorectal tumours demonstrated branch-specific transcriptional alterations, marked by coordinated downregulation of sulphur oxidation and clearance genes (*SQOR*, *ETHE1*, *TST*) and of the cysteine-catabolism genes *CDO1* and *CTH*, and selective upregulation of *SLC7A11* and *AHCY*, whereas *CBS* was not significantly altered. The composite programme score differed across CMS subtypes and was consistently lower in mesenchymal CMS4 tumours than in non-CMS4 tumours across all discovery and validation series. This association remained statistically detectable after adjustment for three global tumour-purity metrics. The programme score showed no statistically detectable independent association with overall, recurrence-free, or disease-free survival. Single-cell deconvolution supported expression across epithelial, stromal, and immune compartments. Direction-aware therapeutic triage prioritised *SLC7A11/xCT* inhibition as an exploratory candidate for preclinical testing.

**Conclusion:**

Sulphur-handling transcriptional architecture is reproducibly linked to CRC molecular taxonomy and is not fully explained by measured tumour purity variation, providing a computational framework for future functional and compartment-resolved investigation of sulphur metabolism in stratified CRC populations.

## 1 Introduction

Colorectal cancer (CRC) ranks among the most prevalent and lethal malignancies globally, yet outcomes remain highly heterogeneous even within clinically defined disease stages. CRC is a molecularly heterogeneous disease in which clinical outcome and therapeutic response vary substantially across transcriptional contexts. One of the most useful frameworks for resolving this heterogeneity is the consensus molecular subtype (CMS) classification, which partitions CRC into four biologically distinct groups and provides a useful framework within which metabolic programmes can be interpreted. In particular, CMS4 is characterised by mesenchymal and stromal biology, whereas CMS2 and CMS3 retain stronger epithelial and metabolic features, making CMS a natural framework within which to examine differentiation-linked metabolic transcriptional states (1, 2).

One such transcriptional programme is sulphur metabolism, which includes processes such as transsulfuration, cysteine handling, and mitochondrial sulphide oxidation. Key components of this network are the hydrogen sulphide (H_2_S)-associated enzymes *CTH* and *CBS*, the sulphide-oxidation and clearance machinery (*SQOR*, *ETHE1*, *TST*, *SUOX*), and the cystine-import transporter *SLC7A11* (also known as *xCT*). Given that the panel includes genes with distinct roles in transsulfuration, sulphide oxidation, cysteine handling, and one-carbon metabolism, its aggregate score should be interpreted as a predefined transcriptional composite rather than a direct measure of H_2_S production, sulphur flux, or enzyme activity. While individual genes in this pathway have been implicated in colorectal cancer, including *CBS* promoting tumour bioenergetics and proliferation, *SLC7A11* mediating resistance to ferroptosis, and *CDO1* being frequently silenced by methylation, the behaviour of the broader sulphur-metabolism programme across tumour-versus-normal tissue, CMS subtypes, tumour purity, and cellular compartments has not been systematically characterised in an integrated human CRC analysis (2–4).

Within this network, two genes serve as particularly informative biological anchors. *CDO1* is a cysteine-catabolism gene that is frequently silenced by promoter methylation in colorectal tumours, making its reduced expression a well-established alteration rather than a novel finding. In contrast, *SLC7A11* is typically upregulated in response to oxidative stress, facilitating cystine import and enhancing redox protection. The contrasting behaviours of these genes suggest that CRC may involve selective rewiring of cysteine and sulphur metabolism, rather than a uniform change in all H_2_S-related genes (4, 5).

A major interpretive challenge in bulk RNA-seq programme analysis is distinguishing genuine transcriptional reprogramming from changes in tumour cellular composition. This is especially pertinent for CMS4, where enrichment of stromal and cancer-associated fibroblast (CAF) components can artificially lower apparent epithelial metabolic gene expression in bulk tissue. Therefore, any association between sulphur-metabolism programmes and molecular subtype should be evaluated using orthogonal tumour-purity estimates and, where possible, supported by compartment localisation from single-cell atlases (1, 6, 7).

In this study, we conducted a multi-cohort transcriptomic analysis of a predefined 14-gene sulphur-handling transcriptional programme in human CRC. Specifically, we investigated whether sulphur-handling transcriptional dysregulation in CRC is uniform or arm-specific; whether it is associated with CMS molecular identity independently of tumour purity; whether it provides independent prognostic information; and whether its transcriptional pattern is associated with molecular features consistent with differentiated epithelial biology. We report that the programme is altered in a functional arm-dependent manner, with coordinated reduction of oxidation/clearance genes alongside selective induction of redox-associated genes such as *SLC7A11*, and that the composite programme score tracks consensus molecular subtype identity.

## 2 Methods

### 2.1 Data sources and cohort assembly

Human CRC gene expression data were obtained from two primary sources. TCGA-colon adenocarcinoma (COAD; *n* = 483 tumours, 41 adjacent normal) and TCGA-rectal adenocarcinoma (READ; *n* = 167 tumours, 10 adjacent normal) were downloaded as STAR-aligned raw read count matrices via the Genomic Data Commons (GDC) data portal (GDC Data Release 37.0).

For microarray-based analyses, six independent HG-U133 Affymetrix CRC cohorts were included in the CMS validation metacohort: GSE39582, GSE14333, GSE17536, GSE17537, GSE33114, and GSE38832. The six-cohort HG-U133 metacohort contained 1,337 input tumours. Of these, 1,187 received a CMS1–4 assignment with nearest template prediction (NTP) false-discovery rate (FDR) ≤ 0.05 and were included in CMS-stratified and meta-analytic analyses; 150 tumours were CMS-unclassified. Cohort-specific analytic sample counts are provided in **Supplementary Tables S1**, **S4**, and dataset selection is summarised in **Supplementary Figure S1**. All cohorts were processed independently; no cross-cohort normalisation was applied prior to subtype classification. Cohort-level sample characteristics including tumour stage, microsatellite instability (MSI) status, KRAS, BRAF, and TP53 mutation status, and tumour location were extracted from the respective supplementary annotations where available (GSE39582) or from TCGA clinical data files (TCGA-COAD/READ). The predefined 14-gene sulphur-handling panel was defined *a priori* as follows: *CTH*, *CBS*, *MPST* (synthesis arm); *SQOR*, *ETHE1*, *SUOX*, *TST* (oxidation/clearance arm); *SLC7A11*, *CDO1*, *CSAD* (cysteine/redox arm); *GOT1*, *GOT2*, *MAT2A*, *AHCY* (methionine/other). Panel composition was fixed before any expression analysis and was not modified based on results.

### 2.2 Differential expression analysis

Tumour-versus-adjacent-normal differential expression was analysed separately in TCGA-COAD and TCGA-READ using edgeR and the limma-voom framework (R/Bioconductor; edgeR v4.8.2; limma v3.66.0). The analysis included all available tumour and adjacent-normal samples within each disease site and treated samples as independent; within-patient correlation for matched tumour-normal specimens was not modelled. Raw STAR counts were filtered with edgeR filterByExpr using tumour/normal group membership, and library-composition factors were estimated by trimmed mean of *M*-values (TMM) normalisation. Counts were transformed with voom using its default normalise method = “none”; quantile normalisation was not applied to the RNA-seq data. A linear model was fitted with tumour/normal status as the primary covariate, followed by empirical-Bayes moderation. Multiple testing correction used the Benjamini–Hochberg procedure; FDR < 0.05 and |log_2_FC| > 0.2 were used as reporting thresholds, while results for all 14 predefined panel genes were retained. Differential-expression results for the full panel are provided in **Table 1** (TCGA-COAD) and **Supplementary Table S2** (TCGA-READ). Colon-versus-rectum consistency was assessed by comparing per-gene log_2_FC estimates across cohorts (**Supplementary Figure S2**). To summarise tumour-versus-normal effects across the two disease sites, per-gene log_2_FC estimates and standard errors from TCGA-COAD and TCGA-READ were combined in a two-cohort random-effects meta-analysis using the *metafor* R package, with the log_2_ fold change as the effect measure, restricted maximum-likelihood estimation of the between-cohort variance, and heterogeneity summarised by Cochran’s *Q* and *I*^2^ (**Figure 1B**, **Supplementary Table S11**). Volcano plots were generated with ggplot2, with sulphur-metabolism panel genes highlighted by direction and significance. This retained the prespecified independent-sample comparison while reporting uncertainty for every panel gene.

**Table 1:** Gene-level tumour-versus-normal differential expression in TCGA-COAD across the 14-gene sulphur-metabolism panel.

Arm	Gene	log_2_FC	SE	Nominal *p*	FDR
Synthesis	CTH	− 0.57	0.140	4.8 × 10^−5^	1 × 10^−4^
Synthesis	CBS	+ 0.20	0.304	0.515	0.565
Synthesis	MPST	− 0.39	0.146	0.008	0.013
Oxidation/clearance	SQOR	− 1.50	0.133	1.1 × 10^−26^	1.4 × 10^−25^
Oxidation/clearance	ETHE1	− 1.55	0.160	9.8 × 10^−21^	8.5 × 10^−20^
Oxidation/clearance	SUOX	− 0.52	0.089	1.1 × 10^−8^	3.7 × 10^−8^
Oxidation/clearance	TST	− 1.70	0.180	1.3 × 10^−19^	1 × 10^−18^
Cysteine/redox	SLC7A11	+ 2.57	0.251	1.2 × 10^−22^	1.2 × 10^−21^
Cysteine/redox	CDO1	− 2.37	0.235	5.6 × 10^−22^	5.3 × 10^−21^
Cysteine/redox	CSAD	+ 0.42	0.131	0.002	0.003
Methionine/other	GOT1	− 0.87	0.117	4.2 × 10^−13^	2 × 10^−12^
Methionine/other	GOT2	+ 0.22	0.093	0.018	0.026
Methionine/other	MAT2A	+ 0.11	0.073	0.131	0.163
Methionine/other	AHCY	+ 1.46	0.148	2.4 × 10^−21^	2.2 × 10^−20^

This table reports results for every predefined panel gene. Differential-expression analysis used edgeR TMM normalisation and limma-voom with Benjamini–Hochberg false-discovery-rate correction. For each gene, log_2_FC, standard error, nominal *p*-value, and FDR are shown. Negative log_2_FC values denote lower transcript abundance in tumour than in adjacent-normal tissue.

**Figure 1:**
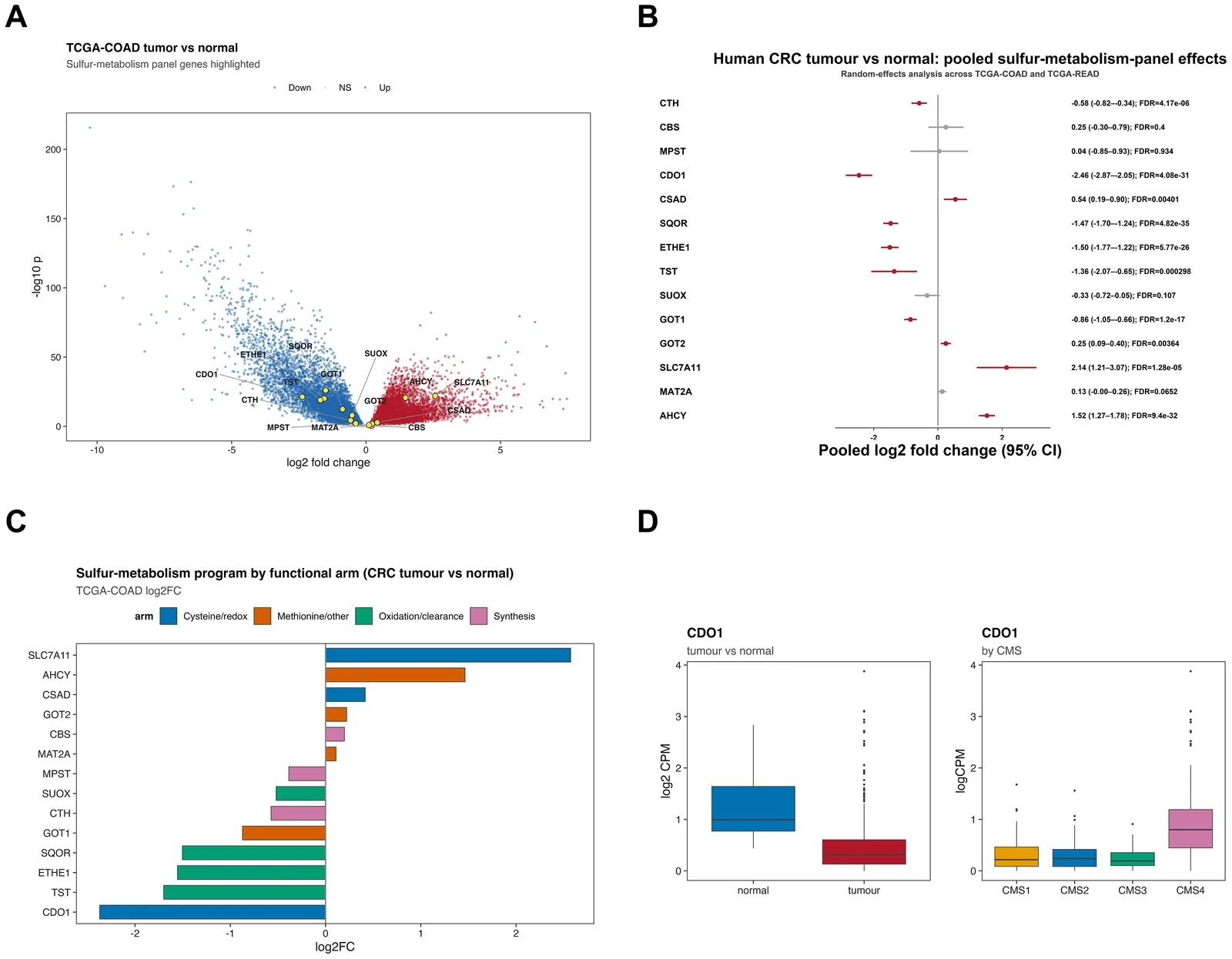
Arm-resolved sulphur-handling transcriptional changes in colorectal cancer. **(A)** Volcano plot of tumour-versus-adjacent-normal differential expression in TCGA-COAD. Genes in the predefined 14-gene sulphur-handling panel are labelled and coloured by direction and statistical significance. **(B)** Random-effects meta-analysis of tumour-versus-normal log2 fold changes across TCGA-COAD and TCGA-READ. Points indicate pooled estimates, and horizontal lines indicate 95% confidence intervals. **(C)** TCGA-COAD tumour-versus-normal log2 fold changes for panel genes grouped by predefined functional arm. **(D)** CDO1 expression in TCGA-COAD adjacent-normal and tumour tissues and across CMS groups in tumours. Boxplots show the median, interquartile range, and whiskers extending to 1.5 × interquartile range; points represent individual samples. Differential expression was estimated using limma-voom, and false-discovery rates were adjusted using the Benjamini–Hochberg method. CMS, consensus molecular subtype; CRC, colorectal cancer; FDR, false-discovery rate.

### 2.3 Programme score computation

A 14-gene sulphur-metabolism programme score was computed per sample as the unweighted mean of row-scaled *Z*-scores across all panel genes, calculated independently within each cohort. *Z*-scores were computed within each cohort over all tumours included in the CMS analyses (tumours only); adjacent-normal samples were not used for *Z*-scoring in CMS analyses. For TCGA-COAD and TCGA-READ, *Z*-scores were derived from *voom*-normalised log-CPM values. For HG-U133 microarray cohorts, *Z*-scores were derived from RMA-normalised log_2_ probe-set-level expression values. Where multiple probe sets mapped to one gene, the probe with the highest mean expression across samples was retained. The programme score was used as a continuous variable in all subsequent association and survival analyses. Functional arm means were computed as the unweighted mean log_2_FC across genes assigned to each arm category. This rule was chosen because the probe set with the highest mean expression across samples is generally the most reliably detected measure of a gene’s transcript abundance; it avoids diluting signal by averaging probe sets with heterogeneous hybridisation properties, and unlike variance-based selection it does not preferentially select probe sets whose high variability may reflect cross-hybridisation. The rule is deterministic, was fixed before downstream analysis, and was applied uniformly across all six HG-U133 cohorts.

### 2.4 CMS classification and molecular subtype analysis

CMS classification of all cohorts was performed using CMScaller v2.0.1, which implements Nearest Template Prediction with predefined CRC CMS templates. For TCGA-COAD, raw RNA-seq counts were supplied with RNAseq = TRUE; for HG-U133 cohorts, quantile-normalised expression matrices were supplied with RNAseq = FALSE. A fixed random seed (set.seed(1)) was applied before each permutation-based classification. Samples without a CMS1–4 assignment at NTP FDR ≤ 0.05 were classified as CMS-unclassified and excluded from subtype-stratified analyses. Per-cohort counts of CMS1–4 and CMS-unclassified samples are reported in **Supplementary Table S4**. Programme scores were compared across CMS1–4 groups by Kruskal–Wallis test. Gene-level CMS-specific mean *Z*-scores were computed in TCGA-COAD and visualised descriptively as a heatmap. Molecular-feature associations (MSI/deficient mismatch repair [dMMR] status, KRAS, BRAF, TP53 mutation, and tumour location) were tested by two-sided Wilcoxon rank-sum tests in GSE39582, with Benjamini–Hochberg (BH) correction across the five features; Δ is the mean score for feature-positive minus feature-negative tumours.

### 2.5 Metacohort meta-analysis

To test reproducibility of the CMS4-versus-non-CMS4 programme-score difference across independent cohorts, a random-effects meta-analysis was performed across the six HG-U133 cohorts using the *metafor* R package (v4.8-0; Viechtbauer, 2010). Within each cohort, an unadjusted linear model estimated the mean difference in programme score between CMS4 and non-CMS4 tumours. Cohort-specific estimates were combined using restricted maximum likelihood. Heterogeneity was summarised using Cochran’s Q and *I*^2^. The primary effect measure was the mean difference in programme-score units, calculated as CMS4 minus non-CMS4. Cohort-specific estimates, pooled results, and heterogeneity statistics are reported in **Supplementary Table S12**.

### 2.6 Tumour purity assessment and confounder control

Three complementary approaches to tumour-purity estimation were evaluated in TCGA-COAD. First, expression-signature-based ESTIMATE scores were computed from the TMM-normalised log_2_-CPM matrix and converted to ESTIMATE-based purity. Second, multiplatform consensus purity estimates (CPE) were obtained from the TCGA pan-cancer purity resource. Third, copy-number-based ABSOLUTE purity estimates were matched from the same resource. Concordance between ESTIMATE-based purity and CPE was assessed by Spearman correlation. The programme score and all continuous covariates, including the ESTIMATE, CPE, and ABSOLUTE purity estimates, were entered in their original scales without further standardisation; purity was entered as a proportion on the 0–1 scale. The CMS4 coefficient therefore represents the adjusted mean difference in cohort-standardised programme-score units between CMS4 and non-CMS4 tumours, and each purity coefficient is expressed per unit increase in the corresponding purity estimate. A clinically adjusted model adding a deconvolution-derived tumour-cell fraction was treated as exploratory. Exact formulas, coding, complete-case *n*, *β*, standard error (SE), 95% confidence interval (CI), and *p*-values are reported in **Supplementary Table S6**.

### 2.7 Survival and prognostic analysis

Prognostic association of the sulphur-metabolism programme score was evaluated across three independent survival endpoints: overall survival (OS) in TCGA-COAD; recurrence-free survival (RFS) in GSE39582; and disease-free survival (DFS) in GSE17536. The sulphur-metabolism programme score was evaluated continuously in Cox proportional-hazards models. TCGA-COAD models were adjusted for age, sex, and categorical pathological stage, followed by additional CMS and ESTIMATE-based purity adjustment. GSE39582 and GSE17536 models incorporated available age, sex, and categorical stage; CMS and purity were not added to the GEO models. Per-gene TCGA-COAD models were adjusted for age, sex, and stage, with Benjamini–Hochberg correction across all 14 panel genes. Kaplan–Meier curves were generated by median programme-score split for visual reference only; these analyses were not used for primary inference. Per-gene Cox models were fitted in TCGA-COAD OS adjusted for age, sex, and stage; FDR correction was applied across all 14 genes. All survival analyses were performed using the *survival* and *survminer* R packages. Results are reported as hazard ratios (HR) with 95% confidence intervals and two-sided *p*-values.

### 2.8 Single-cell deconvolution and compartment localisation

To characterise compartment-associated expression patterns of the predefined sulphur-handling transcriptional programme, BisqueRNA reference-based deconvolution was applied to TCGA-COAD bulk expression matrices using a 2,500-cell donor-balanced single-cell CRC reference derived from GSE178341. GSE178341 was used as a single-cell reference and was not treated as a spatial atlas. Reference labels were retained as tumour-epithelial, CAF/fibroblast, myeloid, lymphoid, and other stromal for deconvolution. Estimated compartment fractions were examined across CMS subtypes (8). Global purity estimates were used in the primary compositional-sensitivity models; a deconvolution-derived tumour-cell fraction was additionally evaluated in a clearly labelled exploratory model. Gene–fraction correlations were interpreted descriptively.

As an independent cross-resource localisation plausibility check, 2,000 donor-balanced primary-tumour cells were sampled in backed mode from the crc.icbi.at atlas (Salcher et al., 2022; 4,264,929 cells, 650 donors, 49 studies). Per-gene, per-compartment mean log1p(CP10K) expression was computed independently in this atlas and in the GSE178341 reference. For this comparison, GSE178341 CAF/fibroblast and other stromal labels were pooled into a common stromal category, yielding four harmonised compartments: tumour-epithelial, stromal, myeloid, and lymphoid. SQOR was unavailable in the sampled GSE178341 matrix, leaving 13 shared genes across four compartments, yielding 52 matched pairs. These aggregated localisation values were compared by Spearman correlation. This cross-resource comparison was interpreted as a localisation plausibility check, not as validation of BisqueRNA fractions in individual TCGA tumours. Tumour-epithelial labels reflect atlas lineage and tissue annotations rather than copy-number-confirmed malignancy calls. Detailed compartment-level expression, harmonisation, and concordance results are provided in **Supplementary Tables S7**-**S9**.

### 2.9 Gene-set enrichment analysis

Preranked enrichment analysis was performed on TCGA-COAD tumour-versus-normal differential-expression statistics using the limma moderated *t*-statistic (log_2_FC/SE) as the ranking metric. Hallmark gene sets were analysed with fgsea v1.36.2 using set.seed(1), minSize = 5, maxSize = 1,000, and the package’s default multilevel algorithm; this was not a specified 10,000-permutation run. GO Biological Process was analysed with clusterProfiler v4.18.4 using minGSSize = 10 and maxGSSize = 500, and Reactome pathways were analysed with ReactomePA v1.54.0. Results are reported as normalised enrichment scores (NES) with BH-adjusted *p*-values. The Hallmark collection contained 50 gene sets and was queried independently from the much larger GO Biological Process and Reactome collections. No alternative ranking statistic was evaluated in this pipeline, so the enrichment results are interpreted using the executed moderated-t ranking only. Selected Hallmark results relevant to CRC biology are presented in **Table 2**; the full Hallmark output is in **Supplementary Table S3** and GO/Reactome context is shown in **Supplementary Figure S6**.

**Table 2:** Representative Hallmark gene sets by normalised enrichment score in TCGA-COAD tumour-versus-normal differential-expression ranks.

Hallmark gene set	NES	FDR
E2F targets	+ 3.97	5 × 10^−49^
MYC targets (v1)	+ 3.63	9 × 10^−44^
G2M checkpoint	+ 3.51	3 × 10^−39^
MYC targets (v2)	+ 3.27	1 × 10^−19^
mTORC1 signalling	+ 2.44	2 × 10^−14^
Fatty-acid metabolism	− 1.93	7 × 10^−7^
Adipogenesis	− 2.02	6 × 10^−10^
Bile-acid metabolism	− 1.90	1 × 10^−5^
Myogenesis	− 2.05	1 × 10^−9^

This table presents selected representative Hallmark gene sets from the pre-ranked gene set enrichment analysis performed on TCGA-COAD tumour-versus-normal differential-expression statistics. Results are summarised by normalised enrichment score (NES) and Benjamini–Hochberg-adjusted false-discovery rate (FDR). Positive NES values indicate enrichment among genes upregulated in tumour, whereas negative NES values indicate enrichment among genes downregulated in tumour.

### 2.10 Direction-aware therapeutic vulnerability triage

To identify pharmacologically actionable nodes within the predefined sulphur-handling panel, TCGA-COAD log_2_FC values for all 14 panel genes were integrated with three external databases in a direction-aware triage screen. Drug–gene interaction records were retrieved from DGIdb 5.0 (Cannon et al., 2024) via the GraphQL API, retaining only interactions with defined interaction types (inhibitor, activator, modulator). Tractability annotations and clinical development-stage data were obtained from the OpenTargets Platform. High-confidence protein–protein association data were retrieved from STRING v12.0 (Szklarczyk et al., 2023) using a combined interaction score threshold of ≥ 0.70. A candidate gene was advanced for prioritisation only when: (i) a pharmacological agent with defined directionality existed; (ii) the direction of proposed pharmacological modulation was compatible with the observed transcript change (i.e., an inhibitor nominated only for upregulated targets, and an activator only for downregulated targets); and (iii) at least one CRC-specific preclinical publication supported the proposed mechanism. Database matches in the absence of directional compatibility or CRC-specific evidence were not advanced. All nominated candidates are experimental triage outputs and do not constitute treatment recommendations; drug sensitivity, therapeutic dependency, or clinical efficacy cannot be inferred from transcript-level data alone (9, 10).

### 2.11 Statistical software and reproducibility

Analyses were performed in R version 4.5.2 using edgeR 4.8.2, *limma* 3.66.0, *fgsea* 1.36.2, *metafor* 4.8-0, *CMScaller* 2.0.1, *BisqueRNA* 1.0.5, *estimate* 1.0.13, *survival* 3.8-6, survminer 0.5.1, *clusterProfiler* 4.18.4, *ReactomePA* 1.54.0, *ggplot2* 4.0.2, and *ComplexHeatmap* 2.26.1. Multiple testing correction used the Benjamini–Hochberg procedure throughout unless otherwise stated. All code and processed data matrices have been deposited in a public repository (Zenodo, https://doi.org/10.5281/zenodo.22731459); the repository contents are described in the Availability of data and materials statement. The study used only publicly available, de-identified datasets; no institutional ethics approval was required.

## 3 Results

### 3.1 CTH and the sulphide-oxidation arm are coordinately rewired in human colorectal cancer

To characterise the predefined sulphur-handling transcriptional programme in human colorectal cancer, we profiled the predefined 14-gene panel in tumour versus adjacent-normal tissue using TCGA-COAD (*n* = 483 tumour, 41 normal; STAR counts, edgeR/limma-voom) and TCGA-READ (*n* = 167 tumour, 10 normal) as independent cohorts. Differential-expression results for the full panel in TCGA-COAD are presented in **Table 1** and visualised as a volcano plot with sulphur-metabolism panel genes highlighted (**Figure 1A**). The pooled forest plot for COAD and READ is shown in **Figure 1B**. Gene-level directions in TCGA-READ were broadly consistent with TCGA-COAD for the major panel effects, although READ estimates were less precise because only 10 adjacent-normal samples were available. READ was therefore interpreted as supportive anatomical replication rather than definitive confirmation (**Supplementary Figure S2**).

CTH was reduced in both cohorts (TCGA-COAD log_2_FC = − 0.57, FDR = 1.03 × 10^−4^; TCGA-READ log_2_FC = − 0.62, FDR = 0.032; **Table 1**). Rather than representing a uniform decrease in sulphur-handling capacity, the data showed an arm-resolved transcriptional pattern (**Figure 1C**, **Table 3**). The oxidation/clearance arm had the largest mean decrease (mean log_2_FC = − 1.32), comprising SQOR (− 1.50), ETHE1 (− 1.55), SUOX (− 0.52), and TST (− 1.70). The synthesis arm declined modestly (mean = − 0.25), with CTH at − 0.57, CBS near-neutral at + 0.20 and nonsignificant, and MPST at − 0.39. The cysteine/redox arm had a small positive mean (+ 0.21): SLC7A11/xCT (+ 2.57, FDR = 1.2 × 10^−21^) and CSAD (+ 0.42) were partly offset by CDO1 (− 2.37). AHCY (+ 1.46) belongs to the methionine/other arm, whose mean was + 0.23. CBS, the synthesis enzyme frequently linked to CRC bioenergetics, was therefore explicitly treated as a near-neutral, nonsignificant exception rather than being folded into the dominant downregulation narrative. Functional-arm means are descriptive summaries, not formal pathway tests.

**Table 3:** Descriptive functional-arm summaries of tumour-versus-adjacent-normal differential expression in TCGA-COAD.

Functional arm	Genes	Mean log_2_FC
Oxidation/clearance	SQOR, ETHE1, SUOX, TST	− 1.32
Synthesis	CTH, CBS, MPST	− 0.25
Cysteine/redox	SLC7A11, CDO1, CSAD	+ 0.21
Methionine/other	GOT1, GOT2, MAT2A, AHCY	+ 0.23

Mean log2 fold changes are descriptive summaries of predefined gene groupings and do not represent formal pathway-level statistical tests.

The marked reduction in *CDO1* expression (log_2_FC = − 2.37; **Figure 1D**) recapitulates a well-established colorectal-cancer-associated alteration and serves as an internal biological concordance check supporting the validity of the analytical pipeline. These arm-resolved findings are more accurately described as a rewiring of cysteine and sulphur handling during malignant dedifferentiation than as a uniform reduction in an H_2_S signal. Transcript-level observations do not permit inference of enzyme activity or H_2_S flux; all conclusions are framed at the level of the transcriptional programme and direction. Collectively, these data indicate that colorectal tumourigenesis is accompanied by a coordinated downregulation of transcripts involved in the sulphide-oxidation and cysteine-catabolism arms alongside upregulation of redox-protective cystine import, a pattern more consistent with loss of the differentiated colonocyte metabolic programme than with a simple reduction in H_2_S biosynthetic capacity.

### 3.2 The sulphur-metabolism programme is strongly associated with CMS molecular subtype, validated across six cohorts, and resolved at gene level

To assess whether the predefined sulphur-handling transcriptional programme was associated with established CRC molecular identity, we calculated a 14-gene programme score as the mean of within-cohort gene-wise *Z*-scores and compared scores across CMS1–4 tumours classified by CMScaller. Programme scores differed across CMS subtypes in TCGA-COAD (Kruskal–Wallis *p* = 6.20 × 10^−20^) and the independent GSE39582 cohort (*p* = 5.11 × 10^−18^; **Figure 2A**). In both cohorts, CMS4 had the lowest programme scores, whereas CMS3 showed comparatively higher scores. Reproducibility was then assessed in six independent HG-U133 cohorts comprising 1,337 input tumours. Of these, 1,187 received CMS1–4 assignments and were included in the meta-analysis; 150 were CMS-unclassified. CMS4 tumours had lower programme scores than non-CMS4 tumours in every cohort. The pooled random-effects difference was − 0.302 programme-score units (95% CI, − 0.37 to − 0.23; *p* = 1.62 × 10^−17^; *I*^2^ = 63%; Cochran’s *Q* = 12.16, *df* = 5, *p* = 0.0326; *k* = 6; **Figure 2B**), a difference in standardised score units rather than an absolute expression difference. Per-cohort programme-score distributions are shown in **Supplementary Figure S3**.

**Figure 2:**
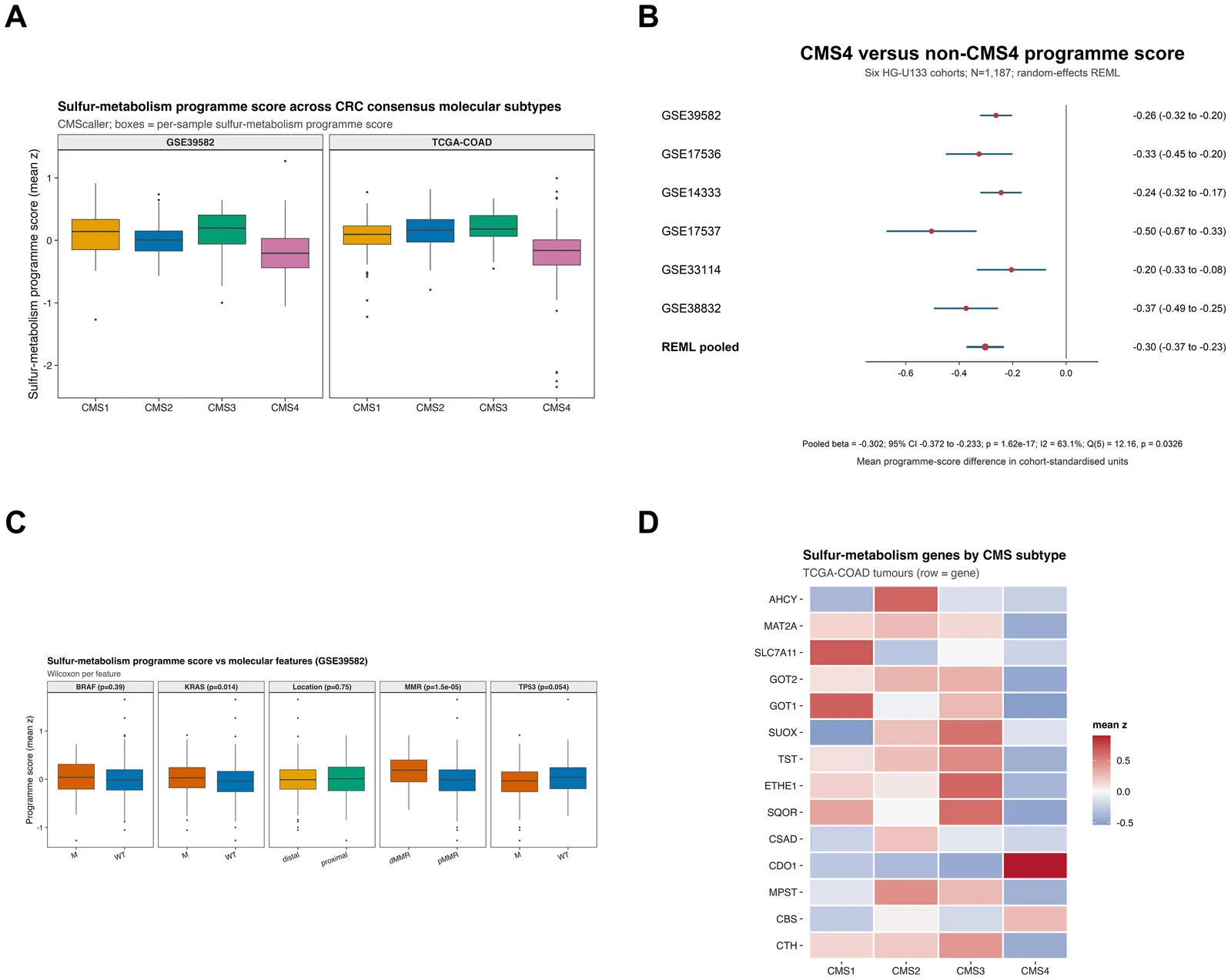
Sulphur-handling programme score across consensus molecular subtypes in colorectal cancer. **(A)** Distribution of the predefined 14-gene programme score across CMS1–4 tumours in TCGA-COAD and GSE39582. Programme scores were calculated as the unweighted mean of within-cohort gene-wise *Z*-scores. Overall group differences were assessed using Kruskal–Wallis tests. **(B)** Random-effects meta-analysis of the mean programme-score difference between CMS4 and non-CMS4 tumours across six HG-U133 cohorts (*k* = 6). The analysis included 1,187 of the 1,337 input tumours that were CMS-classified; 150 tumours were CMS-unclassified. Cohort-specific estimates from unadjusted linear models were pooled using restricted maximum likelihood, and heterogeneity was summarised using Cochran’s *Q* and *I*^2^. The pooled difference was − 0.302 programme-score units (95% CI, − 0.37 to − 0.23; *p* = 1.62 × 10^−17^; *I*^2^ = 63%; *k* = 6), a difference in cohort-standardised programme-score units rather than an absolute expression difference. **(C)** Programme-score associations with selected molecular and clinicopathological features in GSE39582, assessed using two-sided Wilcoxon rank-sum tests with Benjamini–Hochberg correction across five features. **(D)** Mean gene-wise *Z*-scores across CMS subtypes in TCGA-COAD. CMS, consensus molecular subtype; CI, confidence interval; FDR, false-discovery rate.

In GSE39582, programme scores were higher in dMMR than pMMR tumours and modestly higher in KRAS-mutant than KRAS-wild-type tumours after Benjamini–Hochberg correction across the five tested features; associations with BRAF mutation, TP53 mutation, and tumour location were not statistically detectable after correction (**Figure 2C**; **Table 4**; **Supplementary Table S5**). As these were univariable complete-case comparisons, they are interpreted as exploratory molecular-feature associations that may partly reflect underlying CMS composition rather than as feature-specific biomarkers. All CMS and molecular-feature analyses are interpreted as subtype-associated transcriptional biology and do not imply causal directionality. An exploratory unadjusted single-gene comparison of CTH expression by MMR status is shown in **Supplementary Figure S4**.

**Table 4:** Association of the sulphur-metabolism programme score with molecular features in GSE39582.

Feature	*n* positive	*n* negative	Δ score	*p* (Wilcoxon)	FDR
dMMR/MSI	77	459	+ 0.19	1.5 × 10^−5^	7.3 × 10^−5^
KRAS mutant	217	328	+ 0.07	0.014	0.034
BRAF mutant	51	461	+ 0.01	0.394	0.492
TP53 mutant	190	161	− 0.08	0.054	0.09
Distal vs. proximal location	351	232	+ 0.00	0.75	0.75

This table reports selected univariable associations using two-sided Wilcoxon tests. Δ is the mean standardised score for feature-positive minus feature-negative tumours. *p*-values are nominal, and Benjamini–Hochberg-adjusted FDR values are shown and also reported in **Supplementary Table S5**. Positive and negative group *n* values are complete-case counts and therefore vary with feature availability. Associations may partly reflect CMS composition and should be interpreted as exploratory rather than feature-specific effects.

To describe gene-level patterns underlying the programme-score association, we examined mean gene-wise *Z*-scores across CMS1–4 tumours in TCGA-COAD (**Figure 2D**, **Table 5**). *SQOR* and *TST* showed the clearest CMS4-low/CMS3-high pattern among oxidation/clearance genes. *CTH*, a synthesis-arm gene, and *GOT1*, a methionine/other-arm gene, showed a similar descriptive CMS pattern. *SLC7A11* showed a distinct distribution across CMS groups. Formal per-gene inferential testing across CMS subtypes was not performed; therefore, these gene-level patterns are descriptive and hypothesis-generating. They do not establish a causal relationship between CMS identity and programme expression, enzyme activity, H_2_S production, sulphur flux, or therapeutic vulnerability. Taken together, these results show that the sulphur-handling programme score is strongly and reproducibly associated with CMS molecular subtype across seven independent cohorts, with the CMS4-low signal arising primarily from the oxidation/clearance arm and the *SLC7A11* axis operating as a biologically distinct, context-dependent exception.

**Table 5:** Representative per-gene mean *Z*-scores across CMS2, CMS3, and CMS4 tumours in TCGA-COAD.

Gene	CMS2	CMS3	CMS4
SQOR	− 0.02	+ 0.59	− 0.49
TST	+ 0.25	+ 0.47	− 0.43
CTH	+ 0.20	+ 0.40	− 0.45
GOT1	− 0.05	+ 0.25	− 0.54

This table presents representative gene-level mean standardised expression values across CMS2, CMS3, and CMS4 tumours in TCGA-COAD. It illustrates the subtype-resolved transcriptional pattern underlying the sulphur-handling programme association, with several sulphur-handling genes showing lower expression in mesenchymal CMS4 and higher expression in metabolic CMS3. Values are descriptive of transcript abundance across subtypes and should not be interpreted as direct measures of enzyme activity or causal mechanisms underlying subtype differences.

### 3.3 The CMS subtype association is purity-robust, and the programme score has no detectable independent prognostic association

A critical concern for any programme-by-subtype association derived from bulk RNA-seq is confounding by tumour purity and stromal/immune admixture, particularly because CMS4 tumours are stromal-rich. We therefore compared three complementary purity estimates: expression-signature-based ESTIMATE purity, multi-platform CPE, and copy-number-based ABSOLUTE purity. ESTIMATE-based purity and CPE were correlated (Spearman *ρ* = 0.65, *n* = 477; **Figure 3A**), providing evidence of convergence between independent estimation methods. The CMS4-versus-rest programme difference remained negative after clinical adjustment, and after adding ESTIMATE (*p* = 3.65 × 10^−4^), CPE (*p* = 2.60 × 10^−9^), or ABSOLUTE (*p* = 5.09 × 10^−9^) purity, indicating that measured purity differences do not fully account for the subtype association. GSE178341-reference deconvolution showed compartment-fraction differences across CMS subtypes (**Figure 3B**), and CTH was modestly inversely correlated with CAF/fibroblast fraction (*ρ* = − 0.216; **Figure 3C**).

**Figure 3:**
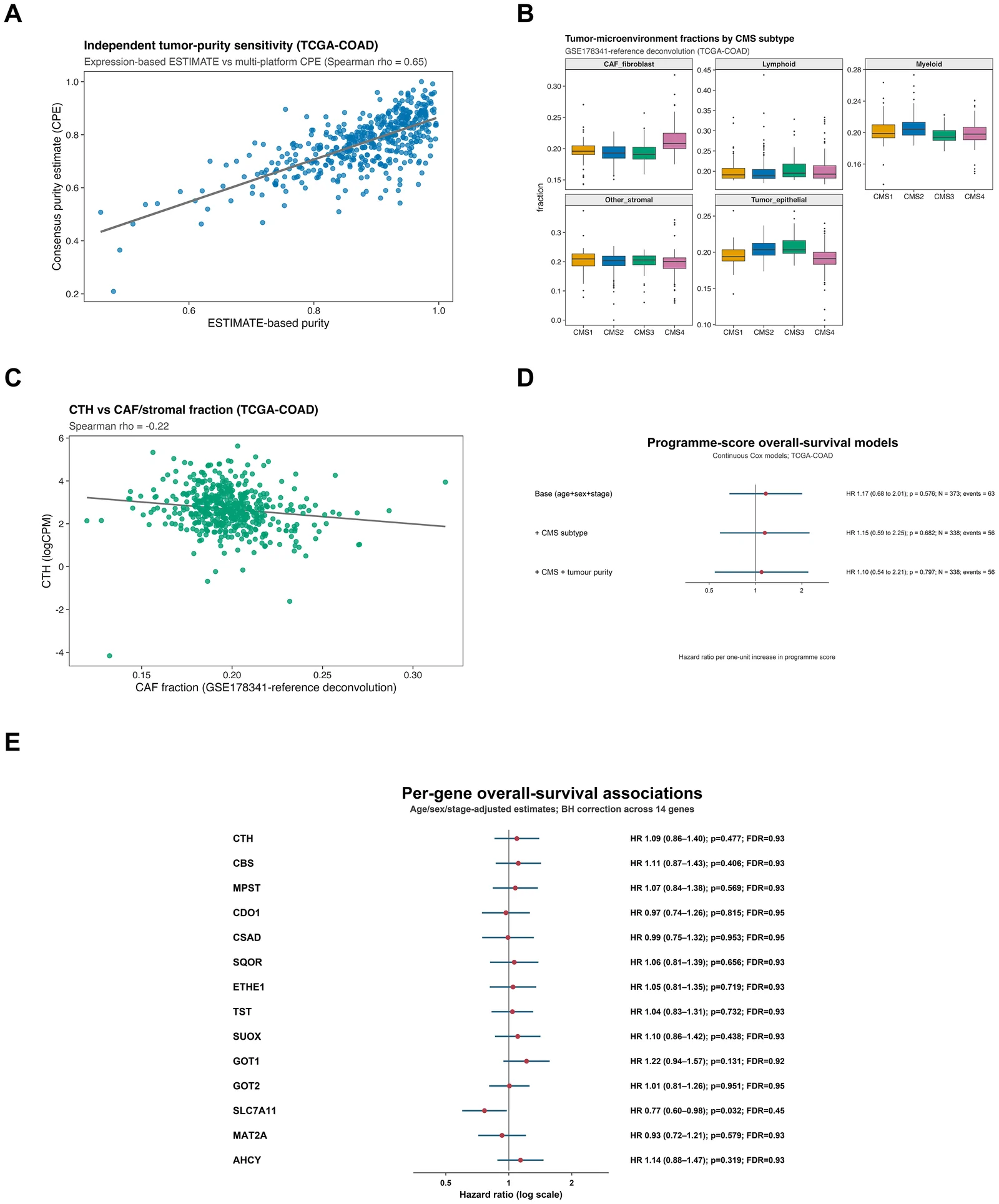
Tumour purity, tumour-microenvironment composition, and survival analyses of the sulphur-handling programme. **(A)** Concordance between ESTIMATE-based and consensus purity estimates (CPE) in TCGA-COAD, assessed by Spearman correlation. **(B)** Estimated tumour-microenvironment compartment fractions across CMS subtypes in TCGA-COAD using GSE178341-reference-based deconvolution. **(C)** Association between CTH expression and estimated CAF/fibroblast fraction in TCGA-COAD, assessed by Spearman correlation. **(D)** Hazard ratios for the continuous programme score in TCGA-COAD overall-survival Cox models adjusted for age, sex, and categorical pathological stage, with additional adjustment for CMS subtype and ESTIMATE-based purity as indicated. Complete-case sample sizes were 373, 338, and 338 participants, with 63, 56, and 56 deaths, respectively. **(E)** Hazard ratios for individual gene-wise *Z*-scores in age-, sex-, and stage-adjusted TCGA-COAD overall-survival Cox models. False-discovery rates were adjusted across the 14-gene panel using the Benjamini–Hochberg method. Deconvolution fractions are model-based estimates and gene–fraction correlations are descriptive. CAF, cancer-associated fibroblast; CMS, consensus molecular subtype; CPE, consensus purity estimate; HR, hazard ratio; OS, overall survival.

With *β* defined as CMS4 minus non-CMS4 (cohort-standardised programme-score units; all covariates on their original scales), the unadjusted estimate was − 0.353 (95% CI, − 0.426 to − 0.280; *n* = 440), and the clinically adjusted estimate was − 0.330 (95% CI, − 0.415 to − 0.245; *n* = 359). In the clinical plus purity models, *β* was − 0.171 with ESTIMATE (95% CI, − 0.265 to − 0.078; *n* = 359), − 0.261 with CPE (95% CI, − 0.345 to − 0.177; *n* = 354), and − 0.252 with ABSOLUTE (95% CI, − 0.335 to − 0.170; *n* = 310). Relative to the unadjusted estimate, attenuation was greatest with ESTIMATE but did not remove the CMS4-associated difference. The exploratory clinical plus deconvolution-derived tumour-cell-fraction model gave *β* = − 0.212 (95% CI, − 0.295 to − 0.129; *n* = 359). Complete-case analysis was used, so sample sizes differ according to covariate availability. Exact formulas, coding, SEs, *p*-values, and sample sizes are reported in **Supplementary Table S6**.

The sulphur-handling programme score showed no statistically detectable association with the evaluated survival endpoints. For each endpoint, survival models were fitted in the subset of participants with complete follow-up and covariate data; this complete-case approach accounts for the reduced analytic samples relative to the parent expression cohorts. Event counts for each model, together with full model specifications and estimates, are reported in **Supplementary Table S10**. In TCGA-COAD, the base model gave HR = 1.17 (95% CI, 0.68–2.01; *p* = 0.576; *n = 373* participants, 63 deaths); the CMS-adjusted model gave HR = 1.15 (95% CI, 0.59–2.25; *p* = 0.682; *n* = 338 participants, 56 deaths); and the CMS-plus-purity model gave HR = 1.10 (95% CI, 0.54–2.21; *p* = 0.797; *n* = 338 participants, 56 deaths) (**Figure 3D**). GSE39582 recurrence-free survival was null (HR = 1.01; 95% CI, 0.61–1.67; *p* = 0.971; *n* = 531 participants, 147 events), whereas the GSE17536 disease-free survival estimate was imprecise (HR = 0.52; 95% CI, 0.25–1.10; *p* = 0.089; *n* = 145 participants, 36 events). No individual gene survived the Benjamini–Hochberg correction across the 14-gene panel (**Figure 3E**); the nominally strongest association was *SLC7A11* (HR = 0.765; 95% CI, 0.599–0.977; nominal *p* = 0.0319; FDR = 0.446) (**Supplementary Figure S5**, **Supplementary Table S10**). Together, these analyses show that the CMS association remains detectable after adjustment for multiple tumour-purity estimates, whereas the programme score itself does not show a detectable independent prognostic association in the datasets examined.

### 3.4 Pathway context and single-cell compartment localisation support a differentiation-state interpretation

To contextualise the programme-level changes within the global transcriptional landscape of CRC and to assess localisation plausibility for the sulphur-handling transcriptional programme across discrete tumour compartments, we combined genome-wide enrichment analysis with single-cell-informed deconvolution in an integrated analysis. Preranked GSEA on TCGA-COAD tumour-versus-normal ranks was performed across three independently curated pathway databases. **Figure 4A** summarises selected CRC-relevant Hallmark pathway axes, **Table 2** presents representative Hallmark results, and **Supplementary Table S3** provides the full Hallmark output; GO Biological Process results are shown in **Supplementary Figure S6A** (clusterProfiler), and Reactome results in **Supplementary Figure S6B**. Across all three resources, CRC tumours are dominated by proliferative programmes (E2F targets NES = + 3.97, FDR = 5 × 10^−49^; MYC targets v1 NES = + 3.63, FDR = 9 × 10^−44^; G2M checkpoint NES = + 3.51, FDR = 3 × 10^−39^; mTORC1 signalling NES = + 2.44, FDR = 2 × 10^−14^; **Table 2**), while the down-regulated sets are enriched for differentiated-tissue metabolic programmes including fatty-acid metabolism (NES = − 1.93, FDR = 7 × 10^−7^), bile-acid metabolism (NES = − 1.90, FDR = 1 × 10^−5^), and adipogenesis (NES = − 2.02, FDR = 6 × 10^−10^). Critically, the sulphur-handling panel declines in parallel with this broader loss of differentiated-epithelial metabolism rather than as an isolated transcriptional event, a pattern consistent with a broader bulk-transcriptomic shift away from differentiated epithelial metabolic programmes.

**Figure 4:**
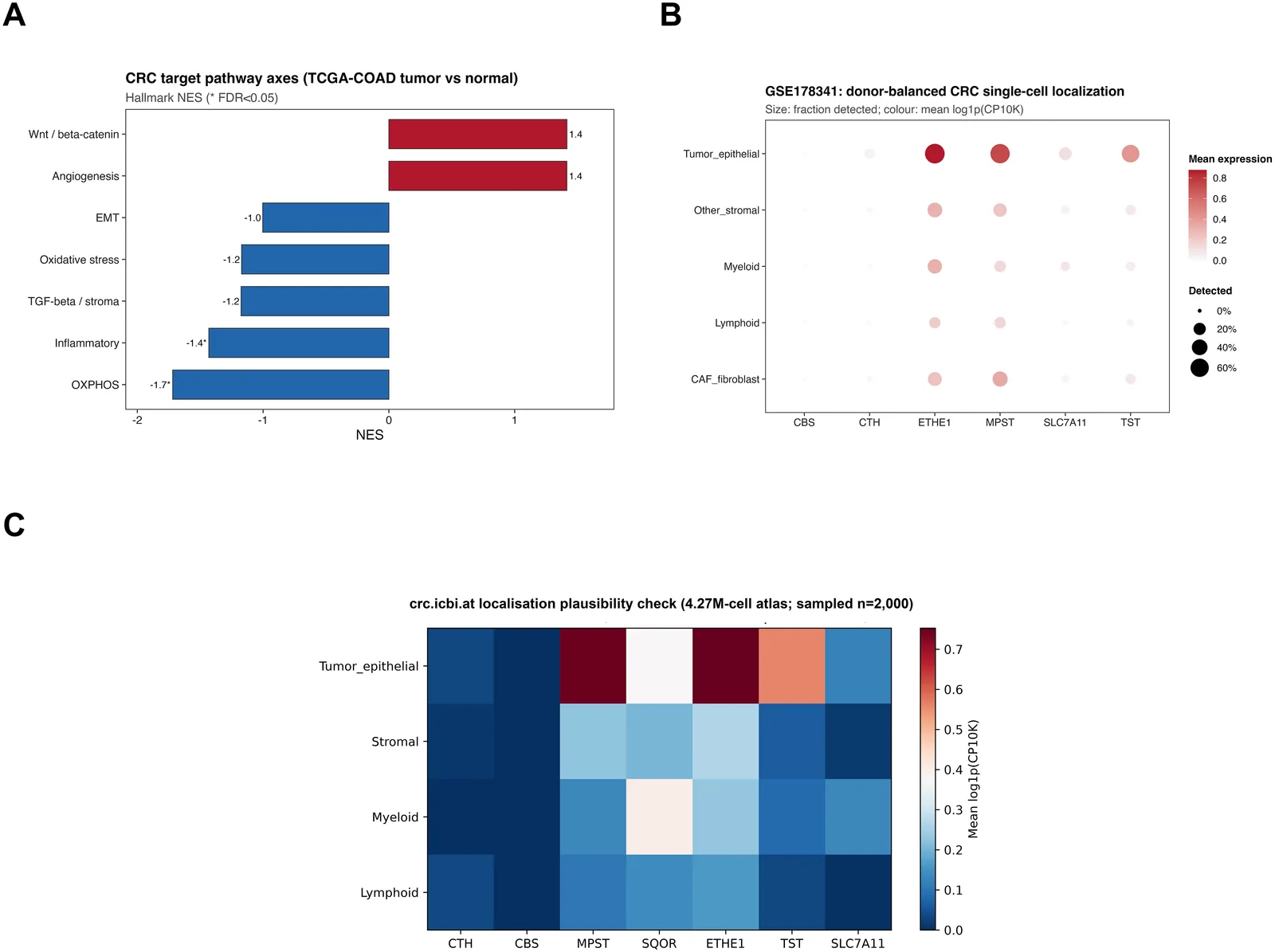
Functional enrichment and compartment-level reference expression of sulphur-handling genes in colorectal cancer. **(A)** Selected Hallmark gene-set enrichment results from TCGA-COAD tumour-versus-adjacent-normal differential-expression ranking. Bars show normalised enrichment scores; asterisks indicate Benjamini–Hochberg-adjusted *FDR < 0.05. **(B)** Donor-balanced GSE178341 single-cell reference expression of selected sulphur-handling genes across annotated cell compartments. Dot size indicates the fraction of cells with detectable expression, and colour indicates mean log1p(CP10K) expression. **(C)** Compartment-level expression patterns in the crc.icbi.at atlas. For cross-resource comparison, GSE178341 CAF/fibroblast and other stromal labels were pooled into a common stromal compartment. SQOR was unavailable in the sampled GSE178341 matrix; the comparison therefore included 13 shared genes across four harmonised compartments, yielding 52 matched pairs. CRC, colorectal cancer; FDR, false-discovery rate; NES, normalised enrichment score.

To assess compartment-associated expression patterns of the sulphur-handling transcriptional programme across tumour cell types, we performed BisqueRNA deconvolution of TCGA-COAD bulk tumours against a 2,500-cell, donor-balanced reference derived from the GSE178341 single-cell CRC reference dataset. Compartment-associated panel-gene expression was examined across tumour-epithelial, CAF/stromal, myeloid, lymphoid, and other stromal fractions (**Figure 4B**). *CTH* showed the largest compartment association with the CAF/stromal fraction (*ρ* = − 0.22), with weaker associations for lymphoid (*ρ* = − 0.12), myeloid (+ 0.16), other stromal (+ 0.08), and tumour-epithelial (+ 0.16) fractions. As a cross-resource localisation plausibility check, we processed 2,000 donor-balanced primary tumour cells from the 4.27-million-cell crc.icbi.at atlas in backed mode, spanning 49 studies assembled independently of the deconvolution reference.

Mean log1p(CP10K) expression was computed independently by gene and harmonised compartment in GSE178341 and crc.icbi.at. GSE178341 CAF/fibroblast and other-stromal categories were pooled into a common stromal category, yielding four harmonised compartments. *SQOR* was unavailable in the sampled GSE178341 matrix, so 13 shared panel genes × four compartments produced 52 matched gene–compartment pairs. The cross-resource Spearman correlation was *ρ* = 0.974 (*p* = 5.10 × 10^−34^; **Figure 4C**); given the non-independence of the 52 gene–compartment pairs, *ρ* is reported as a descriptive concordance measure, and its *p*-value should be interpreted cautiously. Included pairs are listed in **Supplementary Table S7**, localisation values in **Supplementary Table S8**, and the mapping/exclusion ledger in **Supplementary Table S9**. This analysis tests concordance of aggregated localisation patterns between two single-cell resources; it does not validate the accuracy of BisqueRNA fractions in individual tumours. Taken together, the enrichment and localisation results are consistent with a broader shift in differentiated epithelial metabolism, with panel transcripts distributed across multiple compartments rather than confined to one cell type.

### 3.5 Exploratory direction-aware therapeutic vulnerability triage nominates SLC7A11 as the lead preclinical hypothesis

As a tertiary exploratory objective, we performed a direction-aware transcriptomic-to-drug triage screen, joining TCGA-COAD tumour-versus-normal log_2_FC values for all 14 panel genes to live DGIdb 5.0 drug–gene interaction records, OpenTargets tractability annotations, and high-confidence STRING v12 protein associations (score ≥ 0.70). Only *SLC7A11* met all prespecified triage criteria: significant transcript change in a druggable direction, database-supported pharmacological modulation, and CRC-specific preclinical evidence. *CBS* and *MAT2A* did not meet the transcript-direction criterion in this cohort and are retained separately as conditional, literature-anchored hypotheses that would require independent molecular gating (e.g., *CBS* protein/activity, *MTAP* loss) before testing (**Figures 5A, B**, **Table 6**). Database matches alone were not treated as efficacy evidence. As retrospective transcriptomics cannot directly measure pharmacological sensitivity, these nominated agents represent highly prioritised, data-driven candidates for prospective preclinical testing rather than direct clinical treatment recommendations.

**Figure 5:**
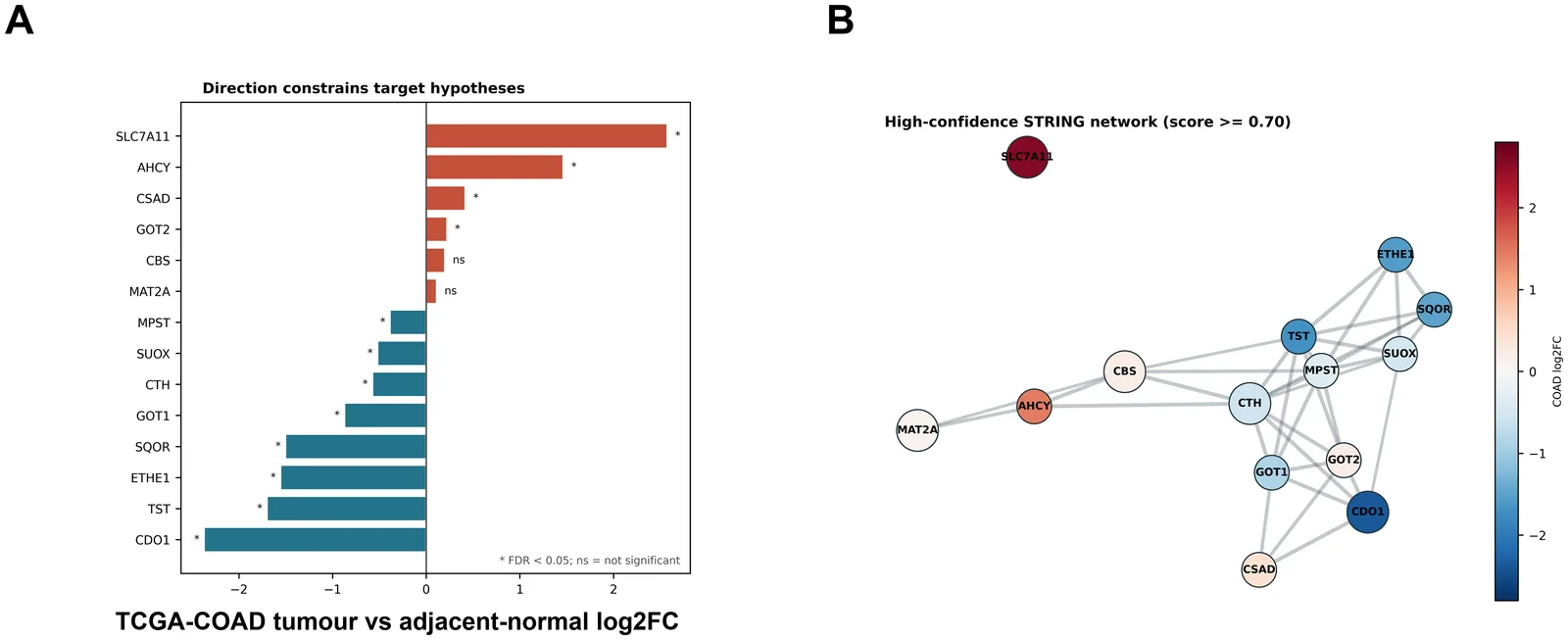
Exploratory direction-aware prioritisation of sulphur-handling therapeutic hypotheses. **(A)** TCGA-COAD tumour-versus-adjacent-normal log2 fold changes for genes in the predefined sulphur-handling panel. Asterisks indicate Benjamini–Hochberg-adjusted FDR < 0.05. **(B)** High-confidence STRING protein-association network for panel genes, restricted to combined interaction scores of at least 0.70 and with colours indicating TCGA-COAD tumour-versus-adjacent-normal log2 fold change. Candidate targets were prioritised using transcript direction, drug–gene interaction directionality, tractability information, and CRC-specific preclinical literature. These analyses are hypothesis-generating and do not establish drug sensitivity, therapeutic dependency, or clinical efficacy. CRC, colorectal cancer; FDR, false-discovery rate.

**Table 6:** Exploratory direction-aware therapeutic triage in colorectal cancer: *SLC7A11* as the sole direction-consistent nomination.

Priority	Target	Example strategy	Interpretation
1	SLC7A11	xCT inhibition (e.g., sulfasalazine/riluzole hypotheses)	Strong transcript direction and CRC preclinical evidence; functional and clinical validation are required.
2	CBS	AOAA prodrug or benserazide	CRC preclinical evidence, but cohort-level transcript is near-neutral; therefore, conditional.
3	MAT2A	AG-270/S095033	Biomarker-gated by MTAP loss; not supported by panel expression alone and not tested here.
–	CDO1/CTH	Restoration concepts	Loss argues against inhibition; no selective, validated clinical strategy nominated.

This table summarises exploratory therapeutic hypotheses generated by integrating TCGA-COAD tumour-versus-normal transcript-direction signals for the 14-gene sulphur-metabolism panel with drug–gene interaction, tractability, and protein-network resources. Candidates were prioritised only when the proposed pharmacological direction was compatible with the observed transcript change and supported by colorectal-cancer-specific preclinical evidence. Sulfasalazine and riluzole are presented as example preclinical xCT-inhibiting tool-compound strategies and not as candidate colorectal cancer treatments; no biomarker-selected clinical efficacy data are available for either agent in colorectal cancer.

*SLC7A11/xCT* (log_2_FC = + 2.57; **Figure 5A**, **Table 6**) was therefore the sole transcript-supported candidate for preclinical functional testing. Sulfasalazine and riluzole, as xCT-inhibiting tool compounds, have sensitised CRC models to cisplatin and radiotherapy through redox/glutathione disruption in preclinical settings, providing directional mechanistic support; however, no biomarker-selected CRC clinical efficacy data exist, and both agents have limited target specificity, so genetic perturbation and orthogonal chemotypes would be required to validate xCT dependence (11).

*CBS* inhibition (AOAA prodrugs, benserazide) ranked second for literature support (12) but remained transcript-conflicted in this cohort because CBS was near-neutral and nonsignificant in tumour-versus-normal analysis (**Figure 5A**, **Table 6**). *MAT2A* inhibition was retained only as an MTAP-loss-gated hypothesis (13), because *MAT2A* itself was not significantly changed and MTAP loss was not tested here (**Table 6**). Conversely, the marked decreases in *CDO1* and *CTH* argue against inhibitory strategies, and any intervention involving those genes requires direct activity and flux measurements before directionality could be inferred (**Figure 5A**, **Table 6**).

The STRING network provides structural context for these prioritisation decisions by showing that *SLC7A11* sits within a broader sulphur-handling network rather than being an isolated outlier (**Figure 5B**). Expression direction and network connectivity alone do not establish dependency, drug sensitivity, or clinical benefit, so this triage should be interpreted as hypothesis-generating only (**Figure 5B**, **Table 6**).

Taken together, this direction-aware triage nominates *SLC7A11/xCT* inhibition as the leading transcript-supported preclinical hypothesis, with CBS and MAT2A strategies retained only as conditional, literature-supported candidates pending direct activity or biomarker confirmation; none of these nominations constitutes a treatment recommendation, and all require independent functional and preclinical validation before any translational claim can be made.

## 4 Discussion

This study shows that a predefined sulphur-handling transcriptional programme is not uniformly altered in colorectal cancer. Oxidation/clearance-arm transcripts are coordinately reduced, synthesis-arm transcripts are largely preserved, and selected redox-associated genes, most prominently SLC7A11, are induced. The composite score of this arm-resolved programme is reproducibly lower in mesenchymal CMS4 tumours and remains evident after adjustment for tumour purity.

This arm-resolved pattern is more consistent with the complexity of sulphur and H_2_S biology described in recent CRC literature than with a simple binary increase-or-decrease model (14, 15).

Across TCGA-COAD and TCGA-READ, the dominant tumour-versus-normal pattern was a coordinated reduction in oxidation/clearance-arm transcripts accompanied by selective upregulation of redox-associated components such as *SLC7A11* and *AHCY*, while *CBS* remained near-neutral. This pattern is best interpreted as selective metabolic reorganisation of cysteine and sulphur handling rather than as a uniform transcriptional surrogate of H_2_S abundance (**Figure 1**), consistent with reviews emphasising that CRC sulphur biology spans endogenous epithelial enzymes, mitochondrial sulphide oxidation, redox buffering, and microbial sulphur inputs that vary independently across contexts (14–16).

A second major finding is the strong and reproducible association between the sulphur-metabolism programme and CRC consensus molecular subtype. In the TCGA-COAD and GSE39582 cohorts, CMS4 tumours showed the lowest programme scores, whereas CMS3 tumours showed comparatively higher scores, and CMS4 tumours had lower programme scores than the combined non-CMS4 group in each of the six HG-U133 validation cohorts (**Figure 2**, **Supplementary Figure S3**), a pattern biologically coherent with the established CMS framework, in which CMS3 reflects epithelial-metabolic dysregulation and CMS4 reflects mesenchymal, stromal, and TGF-beta-associated biology. This association is presented as subtype-associated transcriptional biology, not a causal determinant of CMS identity (2).

Given that stromal and immune admixture can distort bulk transcriptomic signals, particularly in CMS4-like tumours, we tested whether the CMS association survived adjustment for tumour purity. The association persisted after adjustment using ESTIMATE, consensus purity estimates, and ABSOLUTE (**Figure 3**), supporting the interpretation that the pattern is not fully explained by purity alone. Purity estimators remain inferential frameworks rather than direct measurements, so this should be read as robust confounder control rather than complete elimination of compositional bias (17). The compartment-localisation and pathway-enrichment analyses further support an interpretation centered on loss of differentiated epithelial metabolic identity rather than a purely stromal artefact, consistent with broader CRC literature linking subtype structure to shifting relationships among epithelial differentiation, metabolism, and stromal/immune contexture (2). Deconvolution and atlas-validation results should nonetheless remain supportive rather than definitive, as reference-based cell-fraction inference and lineage annotations do not equate to direct cell-state measurement in the analysed tumours.

Across three independent survival endpoints and multiple adjustment levels, the sulphur-handling programme score showed no statistically detectable association with outcome. This is reported as a clean null finding rather than a weak trend, and it does not support positioning the programme as an independent prognostic biomarker. The translational value of this study therefore lies in biologically constrained hypothesis prioritisation rather than in prognosis: the direction-aware triage nominates *SLC7A11/xCT* as the most transcript-supported candidate for preclinical testing within the panel (**Figure 5**), consistent with its established role as a central regulator of cystine uptake, antioxidant defence, and ferroptosis susceptibility in cancer, and its context-dependent dual role highlighted in recent CRC-focussed reviews. *CBS* and *MAT2A* are retained only as conditional, literature-supported hypotheses given their transcript-neutral or untested status in this cohort (18, 19).

That prioritisation is biologically plausible because *SLC7A11* is a central regulator of cystine uptake, antioxidant defence, and ferroptosis susceptibility in cancer, and recent CRC-focussed reviews specifically highlight its context-dependent role as both a tumour-supportive axis and therapeutic vulnerability (20, 21). By contrast, *CBS* and *MAT2A* are more appropriately framed as conditional or context-dependent hypotheses than as validated therapeutic dependencies on the basis of the present transcriptomic evidence alone.

More broadly, these findings fit with emerging evidence that sulphur metabolism in CRC should be understood as a multi-scale system linking tumour-intrinsic transcriptional state, redox adaptation, diet-associated sulphur ecology, and sulphur-metabolising microbial communities (14, 22, 23). That broader context is useful for interpretation, but the current manuscript specifically interrogates human tumour transcriptomes and therefore should not be used to infer microbial sulphur flux, tissue sulphide concentration, or enzyme activity directly (14). CMS is a well-established biological framework for colorectal cancer classification but is not universally implemented as a routine treatment-decision tool; the CMS4 association reported here therefore has biological significance rather than immediate clinical applicability.

This study has several important limitations. First, the central analyses are transcriptomic, and transcript abundance cannot be used to infer enzyme activity, H_2_S production, sulphur flux, or metabolite dependency directly (14). Second, although the CMS association persisted after adjustment using multiple orthogonal tumour-purity frameworks, the study still relies largely on bulk tumour data, so some residual compositional confounding cannot be excluded (24).

Third, the deconvolution and atlas-based localisation analyses provide supportive evidence of compartmentalisation, but they do not substitute for direct single-cell or spatial validation of the malignant compartment in the same samples. Global tumour-purity estimates were used in the primary CMS–programme score compositional-sensitivity models. A deconvolution-derived tumour-cell fraction was additionally included in a clearly labelled exploratory model; this analysis does not eliminate residual compositional confounding or validate estimated fractions in individual tumours. Gene-fraction correlations are descriptive and were not interpreted as evidence of cell-type-specific expression, causal regulation, or biochemical activity.

Fourth, the prognostic analyses were weak or null after multivariable adjustment, and therefore the present data do not justify presenting the sulphur-metabolism programme as an independent prognostic classifier. The prognostic models assumed linear effects of the programme score and did not explore threshold, nonlinear, or subtype-specific (programme × CMS interaction) associations; the null finding therefore applies to independent linear associations and does not exclude all possible prognostic structures. Finally, the therapeutic prioritisation strategy is hypothesis-generating only, because database-supported directionality and network connectivity cannot establish drug sensitivity, target dependency, or clinical efficacy without direct functional testing (20, 21).

In addition, some TCGA participants contributed both tumour and adjacent-normal samples, but the primary differential-expression analysis treated samples as independent and did not explicitly model within-patient correlation; pairing-aware analyses could therefore yield more precise estimates of tumour–normal differences. Moreover, tumour-adjacent normal mucosa was obtained from tumour-bearing colorectum rather than from cancer-free individuals and may consequently be influenced by field effects, local inflammation, altered tissue composition, or physical proximity to the tumour; adjacent-normal reference tissue may consequently not be equivalent to normal colonic tissue obtained from cancer-free individuals. This caveat does not undermine the observed tumour-versus-normal differences but constrains their biological interpretation. TCGA-READ included only 10 adjacent-normal samples, which limits precision and the ability to fully assess technical imbalance; READ was therefore interpreted as supportive anatomical replication rather than as definitive confirmation; accordingly, the strongest tumour-versus-normal evidence derives from colon adenocarcinoma (TCGA-COAD), and tumour–normal effect sizes should be read with this imprecision in mind. Differential-expression estimates may also retain some sensitivity to the chosen RNA-seq normalisation strategy (TMM normalisation followed by voom with normalise method=“none”), so the predefined 14-gene panel is interpreted primarily through effect direction, uncertainty, and consistency across cohorts rather than as an absolute quantitative measure of sulphur-handling activity. The preranked GSEA used the limma moderated *t*-statistic; the Hallmark pathway findings are presented as contextual rather than primary evidence. The programme score is a simple predefined mean-of-*Z* composite; alternative scoring schemes (e.g., PCA-based or ssGSEA) were not explored, and the CMS association could retain some sensitivity to the scoring choice.

CMS-unclassified samples (NTP FDR > 0.05) were excluded from subtype-stratified analyses; these may include intermediate or hybrid tumours, and their exclusion could slightly bias estimates of CMS differences, although per-cohort CMS distributions are fully reported in **Supplementary Table S4**. The random-effects meta-analysis across the HG-U133 validation cohorts includes only six cohorts, so between-cohort heterogeneity estimates are relatively imprecise, and small-sample corrections (e.g., Hartung–Knapp adjustment) were not applied. Molecular feature associations (e.g., MSI, KRAS) in GSE39582 were assessed using univariable tests and may partly reflect underlying CMS structure and stage distribution; they should be interpreted as exploratory associations rather than feature-specific effects. Gene-level CMS patterns (**Figure 2D**, **Table 5**) are descriptive summaries from TCGA-COAD and were not formally tested across subtypes or replicated in independent cohorts; they are used to illustrate, rather than to statistically establish, which genes contribute to the overall programme-score association. Although the CMS framework is a well-established biological classification of colorectal cancer, it is not universally implemented as a routine treatment-decision tool; the CMS4 association reported here therefore speaks primarily to biological stratification rather than to immediate clinical applicability. Accordingly, the predefined sulphur-handling transcriptional programme is currently a biological stratification feature and not a validated clinical assay, prognostic classifier, or treatment-selection biomarker.

## 5 Conclusion

In conclusion, this study supports the conclusion that human CRC exhibits arm-resolved changes in a predefined sulphur-handling transcriptional panel, with lower programme scores in CMS4 than in the other CMS subtypes across multiple cohorts. This association persisted after adjustment for several tumour-purity estimates but was not independently prognostic. The data support sulphur-handling transcription as a subtype-associated systems-biology feature and identify *SLC7A11/xCT* only as a hypothesis for functional preclinical evaluation; they do not establish H_2_S flux, enzyme activity, tumour-cell dependency, or therapeutic efficacy.

## Data Availability

The public datasets analysed in this study are available from the NCBI Gene Expression Omnibus under accession numbers GSE39582, GSE17536, GSE14333, GSE17537, GSE33114, GSE38832, and GSE178341, and from the Genomic Data Commons for TCGA-COAD and TCGA-READ. Custom R and Python scripts, compact metadata, reproducibility files, and processed analysis outputs are deposited in Zenodo, record 22731459, https://doi.org/10.5281/zenodo.22731459.
