# Supplementary material for: A predefined sulphur-handling transcriptional programme is associated with colorectal cancer molecular subtypes
Source: Front Oncol. 2026 Oct 16;16:1976885. doi: 10.3389/fonc.2026.1976885 (PMC13719423; doi:10.3389/fonc.2026.1976885)
Supplement: Supplementary file 1 [file SupplementaryFile1.docx]

**Supplementary Figure S1. PRISMA 2020 Human colorectal cancer dataset identification and selection workflow**

**Supplementary Figure S1. PRISMA 2020 Human colorectal cancer dataset identification and selection workflow.** PRISMA Flow diagram showing identification, deduplication, screening, and inclusion of public human colorectal cancer datasets. Six GEO bulk-expression cohorts, two TCGA cohorts, and two single-cell resources were retained for transcriptomic, CMS, survival, purity, deconvolution, and compartment-localisation analyses. GEO, Gene Expression Omnibus; TCGA, The Cancer Genome Atlas.

**Supplementary Figure S2. Tumour-versus-adjacent-normal differential expression of the sulfur-handling gene panel in TCGA-COAD and TCGA-READ**

**Supplementary Figure S2. Tumour-versus-adjacent-normal differential expression of the sulfur-handling gene panel in TCGA-COAD and TCGA-READ.** Heatmap showing log2 fold changes for the predefined 14-gene sulfur-handling panel in TCGA-COAD and TCGA-READ. Positive values indicate higher expression in tumour relative to adjacent normal tissue, and negative values indicate lower expression in tumour tissue. Differential expression was estimated separately within each disease site using limma-voom. COAD, colon adenocarcinoma; READ, rectal adenocarcinoma.

**Supplementary Figure S3. Cohort-specific distributions of the sulfur-handling programme score across consensus molecular subtypes in six HG-U133 colorectal cancer cohorts**

**Supplementary Figure S3. Cohort-specific distributions of the sulfur-handling programme score across consensus molecular subtypes in six HG-U133 colorectal cancer cohorts.** Boxplots show the predefined 14-gene programme score across CMS1–4 tumours within each cohort. Scores were calculated as the unweighted mean of within-cohort gene-wise z-scores. CMS classification was performed using CMScaller; tumours without a CMS1–4 assignment at NTP false-discovery rate ≤ 0.05 were excluded. Boxplots show the median, interquartile range, and 1.5-times-interquartile-range whiskers; points represent individual tumours. CMS, consensus molecular subtype.

**Supplementary Figure S4. Exploratory comparison of CTH expression by mismatch-repair status in GSE39582**

**Supplementary Figure S4. Exploratory comparison of CTH expression by mismatch-repair status in GSE39582.** Distribution of within-cohort CTH expression z-scores in deficient mismatch-repair (dMMR) and proficient mismatch-repair (pMMR) tumours. The displayed p-value was calculated using a two-sided Wilcoxon rank-sum test and is nominal. Boxplots show the median, interquartile range, and 1.5-times-interquartile-range whiskers; points represent individual tumours. This single-gene analysis is exploratory.

**Supplementary Figure S5. Supporting survival and stage analyses of the sulfur-handling programme in TCGA-COAD**

**Supplementary Figure S5. Supporting survival and stage analyses of the sulfur-handling programme in TCGA-COAD. (A)** Kaplan–Meier overall-survival curves after median dichotomisation of the continuous sulfur-handling programme score. The log-rank p-value is descriptive; median splitting was not used for primary survival inference. **(B)** Hazard ratios for continuous gene-wise z-scores in age-, sex-, and categorical-stage-adjusted Cox proportional-hazards models for overall survival. False-discovery rates were adjusted across all 14 panel genes using the Benjamini–Hochberg method. **(C)** Distribution of the continuous programme score across pathological AJCC stages; groups were compared using a Kruskal–Wallis test. Boxplots show the median, interquartile range, and 1.5-times-interquartile-range whiskers; points represent individual tumours. AJCC, American Joint Committee on Cancer; HR, hazard ratio; OS, overall survival.

**Supplementary Figure S6. Gene Ontology Biological Process and Reactome enrichment analysis of TCGA-COAD tumour-versus-adjacent-normal differential expression**

**Supplementary Figure S6. Gene Ontology Biological Process and Reactome enrichment analysis of TCGA-COAD tumour-versus-adjacent-normal differential expression. (A)** Selected Gene Ontology Biological Process terms and **(B)** selected Reactome pathways derived from pre-ranked enrichment analysis of TCGA-COAD tumour-versus-adjacent-normal differential-expression statistics. Genes were ranked using the limma moderated t statistic. Dot size represents the number of genes contributing to each term, and colour represents the Benjamini–Hochberg-adjusted p-value. GO, Gene Ontology; GSEA, gene-set enrichment analysis.

**Supplementary Table S1. Public human colorectal cancer cohorts and their analytical roles.**

**Note:**
Total sample counts refer to samples available in each source dataset before analysis-specific exclusions. The stated analytical roles indicate the analyses for which each dataset was considered; analytic sample sizes for individual models are reported in the relevant supplementary tables. OS, overall survival; RFS, recurrence-free survival; DFS, disease-free survival; CMS, consensus molecular subtype.

**Supplementary Table S2. Differential expression of the predefined sulfur-handling gene panel in TCGA-READ tumour versus adjacent-normal tissue.**

**Note:**
LogFC represents tumour minus adjacent-normal log2 fold change estimated using limma-voom. SE, standard error; P, nominal two-sided p-value; FDR, Benjamini–Hochberg-adjusted p-value across the 14 predefined panel genes.

**Supplementary Table S3. Hallmark gene-set enrichment analysis of TCGA-COAD tumour-versus-adjacent-normal differential expression.**

**Note:**
Genes were ranked by the limma moderated t statistic. NES, normalised enrichment score; pval, nominal fgsea p-value; padj, Benjamini–Hochberg-adjusted p-value; size, number of genes in the analysed gene set. Positive NES indicates enrichment among genes more highly expressed in tumour relative to adjacent normal, and negative NES indicates enrichment among genes more highly expressed in adjacent normal.

**Supplementary Table S4. CMS classification counts and sulfur-handling panel-gene availability across cohorts.**

**Note:**
CMS classification was performed using CMScaller; tumours with an NTP FDR ≤ 0.05 for a CMS1–4 assignment were classified. CMS-classified n is the sum of CMS1–CMS4 counts. CMS-unclassified tumours were excluded from CMS-stratified analyses. The six HG-U133 cohorts contained 1,337 input tumours, of which 1,187 were CMS classified.

**Supplementary Table S5. Associations of the sulfur-handling programme score with selected molecular and clinicopathological features in GSE39582.**

**Note:**
Two-sided Wilcoxon rank-sum tests compared programme scores between the indicated groups. Delta represents the mean programme score in the feature-positive group minus that in the feature-negative group. FDR values were adjusted using the Benjamini–Hochberg procedure across the five tested features. MMR, mismatch-repair status; pMMR, proficient mismatch repair; dMMR, deficient mismatch repair; WT, wild type; M, mutated.

**Supplementary Table S6. Sensitivity of the TCGA-COAD CMS4-versus-non-CMS4 programme-score association to clinical and compositional adjustment.**

**Note:**
Beta values represent the adjusted mean difference in sulfur-handling programme score for CMS4 minus non-CMS4 tumours. The programme score was calculated as the mean of within-cohort gene-wise z-scores and was entered without further standardisation; continuous purity covariates were entered as original 0–1 proportions. Complete-case sample size varies across models according to covariate availability. ESTIMATE, expression-signature-based purity estimate; CPE, consensus purity estimate; ABSOLUTE, copy-number-based tumour-purity estimate. The model including deconvolution-derived tumour-cell fraction was exploratory.

**Supplementary Table S7. Cross-resource compartment-level expression concordance for sulfur-handling genes across 52 matched gene–compartment pairs.**

**Note:**
Values are independently computed compartment-level mean log1p(CP10K) expression values from GSE178341 and crc.icbi.at. Across the 52 matched gene–compartment pairs, Spearman’s ρ ρ was 0.974 ( p = 5.10 × 10 ^− 34^ ); because genes and compartments contribute repeatedly to the comparison, ρ is interpreted as a descriptive concordance measure and its p-value should be interpreted cautiously. The comparison is a localisation plausibility check and does not validate deconvolution fractions in individual tumours.

**Supplementary Table S8. Donor-balanced single-cell reference expression of sulfur-handling genes across annotated compartments.**

**Note:**
Mean expression is reported as mean log1p(CP10K). Fraction detected is the proportion of sampled cells with non-zero expression. Values are descriptive reference-atlas summaries and do not establish cell-type-specific expression in TCGA-COAD bulk tumours.

**Supplementary Table S9. Harmonisation and exclusion rules for the cross-resource single-cell localisation comparison.**

**Note:**
SQOR was unavailable in the sampled GSE178341 expression matrix and was therefore excluded from the cross-resource comparison. CAF/fibroblast and other-stromal GSE178341 labels were pooled into a common stromal compartment for comparison with crc.icbi.at. The final comparison included 13 shared genes across four harmonised compartments, yielding 52 matched gene–compartment pairs.

**Supplementary Table S10. Continuous Cox proportional-hazards models for the sulfur-handling programme score and individual panel genes, including analytic sample sizes and event counts.**

**Note:**
Hazard ratios for the programme score are reported per one-unit increase in the programme score, whereas hazard ratios for individual genes are reported per one-unit increase in gene-wise z-score. Analytic n denotes the complete-case sample size for the specified model, and events denote deaths for overall survival, recurrence events for recurrence-free survival, and disease-free survival events for disease-free survival. TCGA-COAD programme-score models were adjusted for age, sex, and categorical pathological stage, with additional CMS and ESTIMATE-based purity adjustment where indicated. GSE39582 and GSE17536 models included available age, sex, and categorical stage; CMS and purity were not added to GEO models. Individual-gene TCGA-COAD models were adjusted for age, sex, and stage; FDR values were adjusted across the 14 panel genes using the Benjamini–Hochberg procedure. OS, overall survival; RFS, recurrence-free survival; DFS, disease-free survival; HR, hazard ratio; CI, confidence interval.

**Supplementary Table S11. Random-effects meta-analysis of tumour-versus-adjacent-normal log₂ fold changes for the predefined sulfur-handling gene panel across TCGA-COAD and TCGA-READ.**

**Note:** For each gene, the effect measure was the tumour-minus-adjacent-normal log₂ fold change estimated separately in TCGA-COAD and TCGA-READ. Cohort-specific log₂ fold changes and standard errors were combined using a two-cohort random-effects meta-analysis with restricted maximum-likelihood estimation. k denotes the number of cohorts; Q and Q p-value denote Cochran’s heterogeneity statistic and its p-value; I² denotes the percentage of total variability attributable to between-cohort heterogeneity; and τ^2^ denotes the estimated between-cohort variance. P-values and FDR values refer to the pooled tumour-versus-adjacent-normal contrast, with FDR controlled across the 14 predefined panel genes using the Benjamini–Hochberg procedure.

**Supplementary Table S12. Random-effects meta-analysis of the CMS4-versus-non-CMS4 sulfur-handling programme-score difference across six independent HG-U133 colorectal cancer cohorts.**

**Note:** Cohort-specific effects were estimated as the mean difference in programme score between CMS4 and non-CMS4 tumours, calculated as CMS4 minus non-CMS4. Programme scores were calculated as the mean of within-cohort gene-wise z-scores; therefore, all estimates are expressed in cohort-standardised programme-score units rather than absolute expression units. Cohort-specific estimates were pooled using restricted maximum-likelihood random-effects meta-analysis. The pooled analysis included six cohorts (k = 6) and 1,187 CMS-classified tumours. The pooled estimate was −0.3025 (95% CI, −0.3721 to −0.2329; p = 1.62 × 10⁻¹⁷), with Cochran’s Q = 12.1631 (df = 5, p = 0.03262), I² = 63.07%, and τ^2^	= 0.00444.
